# Mid-term results of titanium–titanium modular neck femoral stem in primary total hip arthroplasty

**DOI:** 10.1186/s12891-024-07370-9

**Published:** 2024-04-02

**Authors:** Kye Young Han, Jinwon Jang, Keong-Hwan Kim

**Affiliations:** 1 Department of Orthopaedic Surgery, Madion Hospital, Anmasan-ro 107, Chuncheon-Si, 24455 Gangwon-Do Republic of Korea; 2 Department of Orthopaedic Surgery, Kangwon National University Hospital, Baengnyeong-ro 156, Chuncheon-Si, 24289 Gangwon-Do Republic of Korea

**Keywords:** Modular neck femoral stem, Offset, Total hip arthroplasty, Junctional problem

## Abstract

**Background:**

Modular neck femoral stems are advantageous because they can accurately restore the ideal hip geometry using various options in terms of offset, length, and version. However, there are concerns regarding junctional problems. Despite several reports on such issues, there is a lack of study on mid- to long-term results of these stems. The current study evaluated the mid-term results of patients who underwent primary total hip arthroplasty using a titanium**–**titanium (Ti–Ti) modular neck femoral stem.

**Methods:**

In total, data on 47 hips (42 patients) that could be followed-up for ≥ 5 years after primary total hip arthroplasty using the Ti–Ti modular neck femoral stem from 2011 to 2015 were reviewed. There were 22 male and 20 female patients, and their mean age was 56.3 (range: 31–76) years. The mean follow-up period was 8 (range: 5–12) years. Functional and radiological outcomes, complications, and reoperations were investigated. In addition, we conducted a comparative analysis of the outcomes between those who underwent surgery using the Ti–Ti modular neck femoral stem and 41 hips (35 patients, 19 males and 16 females) that underwent primary total hip arthroplasty using nonmodular femoral stems as a control.

**Results:**

In all Ti–Ti cases, the mean Harris Hip Scores were 50.6 (range: 6–59) preoperatively and 92.7 (range: 78–99) at the last follow-up (*P* < 0.001). Regarding the neck component’s modularity, straight neck components were used in all Ti–Ti cases, and an anteverted or a retroverted neck was not used in any case. Stem revision was performed in one hip due to aseptic loosening. One hip underwent open reduction and internal fixation due to periprosthetic fracture without stem loosening. There were no cases of osteolysis and periprosthetic joint infection and clinically detectable junctional problems. The stem survival rate, with any stem revision as the endpoint, at 12 years was 96.6%. No significant difference was observed in the functional and radiological outcomes beween the Ti–Ti and nonmodular groups.

**Conclusions:**

The Ti–Ti modular neck femoral stem had comparable results with broadly used nonmodular femoral stems; hence, it can be a reliable option in primary total hip arthroplasty. However, in terms of the modularity itself of the modular neck femoral stem, whether the modular neck femoral stem is useful in uncomplicated primary total hip arthroplasty is unclear.

## Background

Hip offset is a factor that can affect joint reaction force, wear, stability, and longevity in total hip arthroplasty [1–3]. Modular neck femoral stems are advantageous as they can accurately restore the ideal hip geometry [4, 5]. Several authors have reported good clinical outcomes of primary total hip arthroplasty using these stems [6, 7]. In addition, these stems can be helpful in challenging cases such as dysplastic hips, due to various options in terms of offset, length, and version [8].

However, previous studies have reported junctional problems such as corrosion, adverse local tissue reaction, and modular neck fracture [9–12]. In addition, several implants are currently withdrawn from the market [13, 14]. Despite several case reports about junctional problems, studies assessing the mid- to long- term results of primary total hip arthroplasty using the modular neck femoral stem, as compared with broadly used cementless nonmodular fermoral stems, are lacking.

The current study investigated the mid-term (at least 5 years) results of a patient group who underwent primary total hip arthroplasty using the titanium**–**titanium (Ti–Ti) modular neck femoral stem that was used before withdrawal.

## Materials and methods

The current study was approved by the Institutional Review Board of our institution. The electronic medical records and radiographic data of the patients who underwent primary total hip arthroplasty using a highly porous tantalum trabecular metal acetabular cup (Continuum Acetabular System; Zimmer, Warsaw, IN, the USA) and a Ti–Ti modular neck femoral stem (Kinectiv Technology; Zimmer, Warsaw, IN, the USA) from April 2011 to January 2015 were reviewed. Of 72 hips, 35 were lost to follow-up within 5 years. Finally, 47 hips (42 patients, 22 males and 20 females) who were followed-up for ≥ 5 years were included in this analysis. None of the patients presented with procedure-related complications and required stem reivision within 5 years.

Since a recall issue with the modular neck femoral stem in 2015, cementless nonmodular femoral stems have been primarily used in primary total hip arthroplasty at our institution. As a control, cases that underwent primary total hip arthroplasty using cementless nonmodular femoral stems (Ecofit; Implantcast GmbH, Germany and ML taper, Zimmer, Warsaw, IN, the USA) until 2018 were reviewed. Altogether, 41 hips (35 patients, 19 males and 16 females) who were followed-up for ≥ 5years were included as controls.

The surgery was performed by one senior author using the modified Watson-Jones approach in the lateral position. After the procedure involving the acetabular cup, the procedure involving the femoral stem was performed. Stem size and neck offset were selected by evaluating leg length and stability intraoperatively, referred to planned stem size and neck offset using the preoperative template. The default version of the neck component was straight. However, anteverted or retroverted necks were considered if there were concerns about stability based on the examination after the trial insertion, intraoperatively.

Functional and radiological outcomes were investigated postoperatively. For functional evaluation, the Harris Hip Score (HHS) was examined and compared before and after surgery [15]. For radiological evaluation, the inclination and anteversion of the acetabular cup [16, 17], leg length discrepancy, stem subsidence, fixation of the stem and cup [18, 19], and osteolysis around the stem and cup according to the specific zones were evaluated on plain radiography [20, 21]. The inclination and anteversion of the cup and leg length discrepancy were measured on immediate postoperative hip anteroposterior radiography. Leg length discrepancy was defined as the difference in the distance between the level of the lower margin of the tear drop and the level of the apex of the lesser trochanter at both sides. Stem subsidence, fixation of the stem and cup, and osteolysis were evaluated in the last follow-up. To investigate the junctional problems, the symptoms and plain radiograpy and computed tomography (CT) scan were evaluated. Although CT was not routinely performed, in cases in which the CT scan of the abdominopelvic area or lower extremity was performed for medical evaluation at other departments in our institution during the follow-up period, abnormal findings associated with junctional problems such as fluid collection and cyst formation around the hip joint were evaluated [22]. If revision surgery was performed during the follow-up period, the cause of revision surgery was investigated.

Statistical analysis was performed using the Statistical Package for the Social Sciences software version 21.0 (SPSS Inc., Chicago, IL, the USA). For continous variables, t-test or nonparametric test was performed depending on whether the data have a normal distribution. Chi-square or Fisher’s exact test was used to examine for frequencies. P value of < 0.05 was considered statistically significant. In addition, the stem survival rate was investigated via Kaplan–Meier survival analysis.

## Results

Regarding the demographic characteristics of the patients, no significant difference was observed between the Ti–Ti and the nonmodular groups (Table 1) [23–25]. The mean follow-up periods were 8 (range: 5–12) yeas and 6.4 (range: 5–8) years for the Ti-Ti and nonmodular groups, respectively.

**Table 1:** Demographic data

	Ti–Ti modular neck femoral stem (*n* = 47)	Nonmodular femoral stem (*n* = 41)	P value
Age (years)	56.3 ± 11.4	60.6 ± 12.8	0.080
Sex (M/F)	22 (26 hips)/20 (21hips)	19 (23 hips)/16 (18 hips)	0.942
Height (cm)	163.7 ± 8.8	160.1 ± 9.0	0.062
Weight (kg)	62.7 ± 11.9	63.1 ± 11.3	0.795
Body mass index (kg/m^2^)	23.3 ± 2.8	24.6 ± 3.7	0.065
American Society of Anesthesiologists classification	1.6 ± 0.7	1.8 ± 0.7	0.084
Charlson comorbidity index	0.7 ± 1.2	0.5 ± 0.9	0.475
Koval grade	1.6 ± 1.3	1.6 ± 1.6	0.653
Causes of total hip arthroplasty			0.744
Osteonecrosis	31	27	
Osteoarthritis	12	10	
Femoral neck fracture	2	4	
Rheumatoid arthritis	1	0	
Giant cell tumor	1	0	

Continuous variables are evaluated as the mean value ± standard deviation

Ti–Ti; titanium**–**titanium

In all Ti–Ti cases, straight neck components were used, and an anteverted or a retroverted neck was not used in any case. The neck offset was selected according to the patient’s own hip geometry (23 A, 16 B, 5 C, 1 E, 1 G, and 1 J). For the bearing surface, ceramic-on-ceramic was used in 36 cases and ceramic-on-highly cross-linked polyethylene in 11 cases, and the head sizes were 36, 32, and 28 mm in 23, 23, and 1 case, respectively. The mean inclination and anteversion of the cup were 43.5° (range: 32°–59°) and 23° (range: 10°–38°).

Regarding the posopoperative leg length discrepancy, no significant difference was observed between the Ti–Ti and nonmodular groups [1.62 (range: 0–8) vs. 1.66 (range: 0–9), *P* = 0.834]. No significant difference in other radiological outcomes also noted between the two groups. (Table 2). All acetabular cups were obtained via stable fixation with bone ingrowth. There was no case of osteolysis in both groups.

**Table 2:** Clinical outcomes

	Ti–Ti modular neck femoral stem (*n* = 47)	Nonmodular femoral stem (*n* = 41)	P value
Stem fixation			1.000
Bony ingrowth	45	39	
Stable fibrous	1	1	
Unstable	1	1	
Stem subsidence (mm)	3.5 (range: 2–9, *n* = 6)	2.8 (range: 2–4, *n* = 4)	1.000
Leg length discrepancy (mm)	1.62 (range: 0–8)	1.66 (range: 0–9)	0.834
Postoperative HHS	92.7 (range: 78–99)	91.7 (range: 75–98)	0.170
Stem revision	1	2	0.596

Ti–Ti; titanium**–**titanium, HHS; Harris Hip Score

In the Ti–Ti group, the mean preoperative and postoperative HHS were 50.6 (range: 6–59) and 92.7 (range: 78–99) (*P* < 0.001). There was no significant difference in postoperative HHS between the two groups (Table 2). In both groups, none of the patients developed dislocation and periprosthetic joint infection. In addition, there were no clinically detectable junctional problems based on symptoms and plain radiography and CT scan results in the Ti–Ti group. In 18 (19 hips) patients, CT scan of the abdominopelvic area or lower extremity was performed at other departments during the follow-up period. Further, there was no periarticular pathologic finding around the prosthesis indicating junctional problems.

**Fig. 1:**
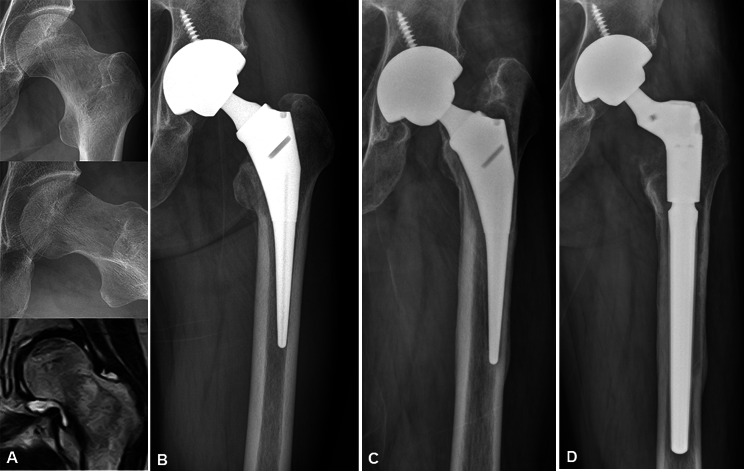
A 52-year-old male patient underwent total hip arthroplsty for osteonecrosis of the femoral head of left hip (**A**, **B**). At 8 years after the surgery, stem revision was performed due to aseptic loosening of the stem (**C**, **D**)

In one hip, stem revision was performed due to aseptic loosening at 8 years after surgery in the Ti–Ti group (Fig. 1). In addition, open reduction and internal fixation was conducted for proximal femoral periprosthetic fracture without stem loosening in one case. In the nonmodular group, stem revision was performed in two cases at 6 years after surgery due to aseptic loosening and proximal femoral periprostehtic fracture.

**Fig. 2:**
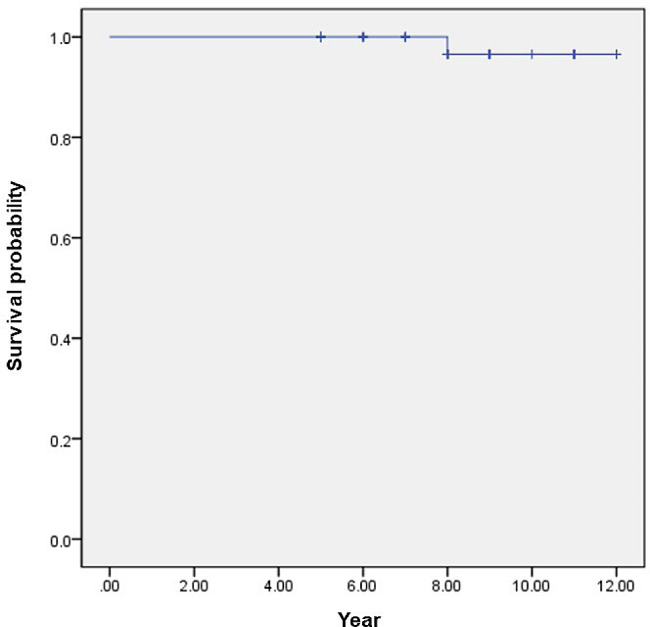
Kaplan–Meier survival curve of the Ti–Ti modular neck femoral stem with any stem revision as the end point

The stem survival rate of the Ti–Ti modular neck femoral stem, with any stem revision as the endpoint, at 12 years was 96.6% (Fig. 2). In the nonmodular femoral stems, the stem survival rate, with any stem revision as the endpoint, at 8 years was 93.8%.

## Discussion

Despite the theoretical advantages, there are concerns about junctional problems between the modular neck component and the stem body in the modular neck femoral stems [26]. However, mid- to long-term follow-up studies on the Ti–Ti modular neck femoral stem are lacking. The current study reported the results of our patients who underwent primary total hip arthroplasty using the modular neck femoral stem, with a mean follow-up of 8 years. The results were comparable, and there were no clinically detectable junctional problems.

In terms of the modularity of total hip arthroplasty, the research results on the head-neck taper were relatively greater than that on the modular neck [27]. Corrosion according to metal combination in the head-neck taper can occur over time. However, the parameters are different for similar and different metal combinations [28]. In addition, several studies have reported that mixed metal combinations in the head-neck taper are more susceptible to corrosion than similar metal combinations [29, 30]. Similarly, several authors have reported an increase in serum cobalt and chromium metal ions in cases that used a combination of titanium stem and cobalt–chromium neck, and based on these phenomenon, the limited use of this type of modular neck femoral stem was recommended [31–33]. According to existing literature, the combination of titanium stem and cobalt–chromium neck was commonly used in failed cases [10, 33]. There are only a few cases of junctional problems in the Ti–Ti modular neck [9, 11]. In addition, several clinical studies have shown that the Ti–Ti modular neck femoral stem has good clinical results [6, 7, 34]. This finding is in accordance with our findings. Although there are various factors influencing junctional problems [27], the results might have been influenced by the metal combination between the stem and modular neck. However, additional comparative studies should be conducted to validate this notion.

Considering the neck components used in this study group, the usefulness of the modularity itself of the modular neck femoral stem in cases of uncomplicated primary total hip arthroplasty has not been confirmed. The offset and length of the neck component were planned according to the preoperative template, and the neck component was finally determined with consideration of length, offset, and stability based on the intraoperative findings. In the current study, the version neck was not used. In all hips, the straight neck was utilized, and the A or B components were applied in 80% of the hips. In challenging cases such as patients with dysplastic hip accompanied by complex deformity, various modular neck femoral stems are more useful [35]. Sakai et al. conducted a comparative study on the modular neck and nonmodular neck in patients with developmental dysplasia of the hip who underwent total hip arthroplasty [8]. Results showed that the modular neck group had better clinical and radiological findings.

The current study had several limitations. First, it was a retrospective case series with a small number of cases. Second, all asymptomatic junctional problems at the modular neck junction were challenging to rule out, although a comprehensive clinical and radiological analysis including CT scan was performed. Additional metal ion studies and retrieval studies should be conducted to have a better understanding of these issues.

## Conclusions

The Ti–Ti modular neck femoral stem had comparable results with broadly used nonmodular femoral stems; hence, it can be a reliable option in primary total hip arthroplasty. However, in terms of the modularity itself of the modular neck femoral stem, whether the modular neck femoral stem is useful in uncomplicated primary total hip arthroplasty is unclear.

## Data Availability

The datasets used and/or analyzed during the current study are available from the corresponding author on reasonable request.

## Abbreviations

Ti–Ti: Titanium–Titanium
HHS: Harris Hip Score
CT: Computed tomography
